# A Reverse Genetics Platform That Spans the Zika Virus Family Tree

**DOI:** 10.1128/mBio.02014-16

**Published:** 2017-03-07

**Authors:** Douglas G. Widman, Ellen Young, Boyd L. Yount, Kenneth S. Plante, Emily N. Gallichotte, Derek L. Carbaugh, Kayla M. Peck, Jessica Plante, Jesica Swanstrom, Mark T. Heise, Helen M. Lazear, Ralph S. Baric

**Affiliations:** a Department of Epidemiology, Gillings School of Global Public Health, University of North Carolina, Chapel Hill, North Carolina, USA; b Department of Microbiology and Immunology, School of Medicine, University of North Carolina, Chapel Hill, North Carolina, USA; c Department of Genetics, School of Medicine, University of North Carolina, Chapel Hill, North Carolina, USA; d Department of Biology, University of North Carolina, Chapel Hill, North Carolina, USA; Columbia University College of Physicians & Surgeons

## Abstract

Zika virus (ZIKV), a mosquito-borne flavivirus discovered in 1947, has only recently caused large outbreaks and emerged as a significant human pathogen. In 2015, ZIKV was detected in Brazil, and the resulting epidemic has spread throughout the Western Hemisphere. Severe complications from ZIKV infection include neurological disorders such as Guillain-Barré syndrome in adults and a variety of fetal abnormalities, including microcephaly, blindness, placental insufficiency, and fetal demise. There is an urgent need for tools and reagents to study the pathogenesis of epidemic ZIKV and for testing vaccines and antivirals. Using a reverse genetics platform, we generated six ZIKV infectious clones and derivative viruses representing diverse temporal and geographic origins. These include three versions of MR766, the prototype 1947 strain (with and without a glycosylation site in the envelope protein), and H/PF/2013, a 2013 human isolate from French Polynesia representative of the virus introduced to Brazil. In the course of synthesizing a clone of a circulating Brazilian strain, phylogenetic studies identified two distinct ZIKV clades in Brazil. We reconstructed viable clones of strains SPH2015 and BeH819015, representing ancestral members of each clade. We assessed recombinant virus replication, binding to monoclonal antibodies, and virulence in mice. This panel of molecular clones and recombinant virus isolates will enable targeted studies of viral determinants of pathogenesis, adaptation, and evolution, as well as the rational attenuation of contemporary outbreak strains to facilitate the design of vaccines and therapeutics.

## IMPORTANCE

Viral emergence is a poorly understood process as evidenced by the sudden emergence of Zika virus in Latin America and the Caribbean. Malleable reagents that both predate and span an expanding epidemic are key to understanding the virologic determinants that regulate pathogenesis and transmission. We have generated representative cDNA molecular clones and recombinant viruses that span the known ZIKV family tree, including early Brazilian isolates. Recombinant viruses replicated efficiently in cell culture and were pathogenic in immunodeficient mice, providing a genetic platform for rational vaccine and therapeutic design.

## INTRODUCTION

Zika virus (ZIKV), a member of the *Flavivirus* genus and closely related to dengue virus (DENV), was first discovered in 1947 in Uganda (1) and until recently had been responsible for only sporadic human infections in Africa and Asia (2). In the past decade, however, ZIKV has emerged into new areas, including a large outbreak in Micronesia in 2007, where it is estimated that 73% of the population was infected within a 4-month period (3), followed by a 2013-2014 outbreak in French Polynesia and subsequent spread throughout Oceania (4). The ZIKV outbreak in the South Pacific is thought to be the source of virus introduced to Brazil, supported by the close genetic relationship of South Pacific and epidemic Latin American strains (5). The first ZIKV cases in Brazil were reported in early 2015, with the outbreak initially concentrated in the northeastern region of the country (6, 7). Although early case reports were consistent with the self-limited febrile illness observed in previous outbreaks, a surge in cases of microcephaly was reported in the northeastern region of Brazil in the fall of 2015 (8). Thereafter, a growing body of molecular, immunologic, and epidemiological evidence has demonstrated a causal role for ZIKV infection in microcephaly as well as a spectrum of other neurodevelopmental defects, now collectively referred to as “congenital Zika syndrome” (9). To date, it is unknown why transplacental transmission and teratogenicity have been observed during the current ZIKV epidemic in Latin America but not in previous outbreaks. Furthermore, this epidemic has revealed a role for sexual transmission in the spread of ZIKV, a transmission mode not reported for other flaviviruses (10). While some have speculated that genetic changes in the virus could be responsible for new pathogenic phenotypes, testing this hypothesis would be aided by a tractable reverse genetics system to generate panels of isogenic mutants of proposed viral determinants of pathogenesis.

Although cDNA-based infectious clones (ICs) have been generated for other flaviviruses, including West Nile virus, yellow fever virus, DENV, and ZIKV (11–17), flavivirus reverse genetics systems can be more challenging than those for many other viruses, because of sequence instability in bacterial vectors (18). Recent efforts to generate ZIKV recombinant viruses have resulted in different cloning strategies, all of different ZIKV strains, that have used DNA plasmids with introns or as multipiece systems designed to overcome these fundamental stability issues (15–17, 19).

Here, we have developed a panel of ZIKV infectious clones, patterned after DENV and coronavirus strategies (20–22). The ZIKV panel includes three allelic variants of a historical African strain (MR766) as well as contemporary outbreak strains from French Polynesia (H/PF/2013) and early epidemic Brazilian strains (SPH2015 and BeH819015), enabling experimental testing of viral determinants that distinguish the current ZIKV epidemic from earlier outbreaks. In the process of generating clones of the two Brazilian strains, we identified sequence abnormalities that impacted virus viability. Phylogenetic analysis of currently available full-length genomes suggests that two clades of ZIKV are circulating in Brazil (5). We generated molecular clones and recovered recombinant viruses representing early isolates from both Brazilian ZIKV clades.

Based on our previous experience with DENV infectious clones (20–22), we were able to partition toxic regions of the ZIKV genome into stable plasmid subclones. Furthermore, we used nonpalindromic restriction endonuclease sites naturally occurring in the ZIKV genome, allowing directional ligation of digested subgenomic fragments into full-length cDNAs from which full-length infectious transcripts can be synthesized *in vitro*. The resulting ZIKV recombinant viruses grow to similar peak titers as their parental isolate viruses and are recognized by cross-reactive DENV monoclonal antibodies (MAbs) but not DENV serotype-specific MAbs. Recombinant viruses were virulent in *Ifnar1*^−/−^ × *Ifngr1*^−/−^ C57BL/6J mice, though slightly attenuated compared to natural isolate viruses. Interestingly, there was a large difference in murine pathogenesis between the two Brazilian recombinant viruses, driven by one or more of 6 amino acid differences that distinguish the two strains. This highly tractable genetic platform will be useful for evaluating viral determinants of ZIKV pathogenesis and for the development and testing of interventions to combat ZIKV disease.

## RESULTS

### Complete genomic sequence determination of multiple ZIKV strains.

**FIG 1:**
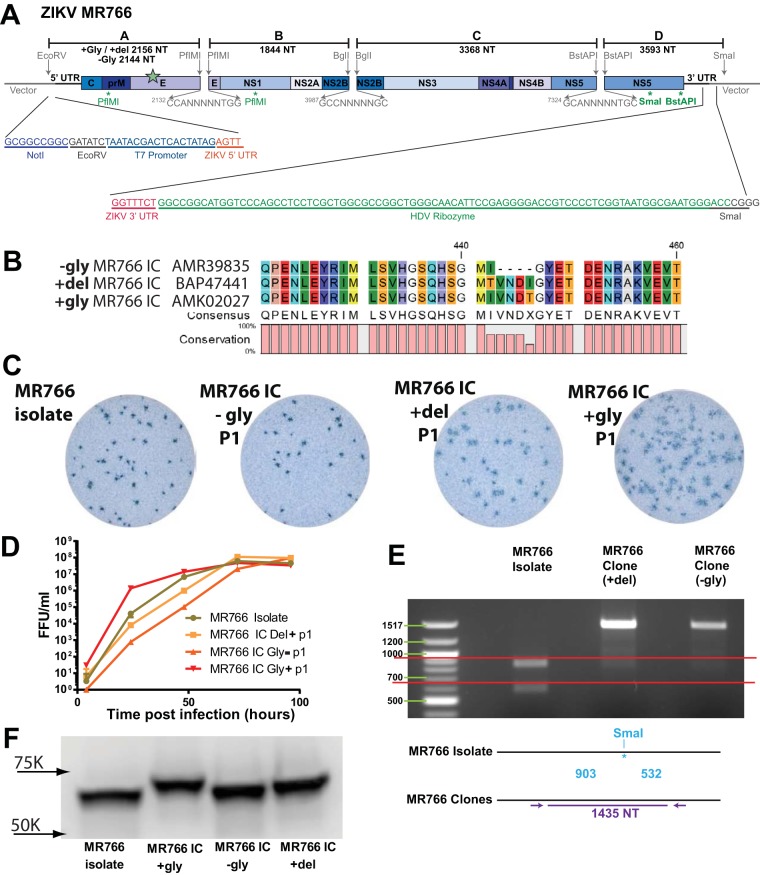
(A) Schematic diagram of ZIKV MR766 infectious clone. The genome of the virus is divided into 4 fragments using the diagrammed restriction endonucleases and cloned into high-copy-number vectors. A T7 promoter is placed before the first nucleotide of the ZIKV genome, and a hepatitis delta virus ribozyme is placed after the final genomic nucleotide for RNA stability. Four restriction endonuclease sites shown beneath the genome (two PflMI, one SmaI, and one BstAPI) were removed using synonymous changes. Sizes of each fragment are shown, and the larger size is listed along with the location of the N154 allelic mutations (green star). (B) Amino acid sequences of the 3 allelic MR766 variants in the region of the N154 glycosylation site on E. Numbers (440 and 460) correspond to amino acid positions in the complete ZIKV genome. (C) Virus focus images on Vero-81 cells. (D) Growth curves of MR766 isolate and 3 infectious clones done at 37°C. MR766 +gly IC had significantly higher early growth than the other 3 strains by 2-way analysis of variance with Tukey’s test. (E) After reverse transcriptase-PCR, viral amplicons were subjected to SmaI digestion; only the DNA from the natural isolate was cut. (F) Western blot of E protein from MR766 isolate and 3 infectious clones performed using the pan-flavivirus MAb 4G2. Size differences of the E protein are shown based on their sequence and glycosylation status.

Constructing an infectious clone requires knowledge of the complete viral genome sequence. However, many early ZIKV sequences deposited in GenBank annotated as being a “complete genome” were actually incomplete (accession numbers KU729218, KU707826, KF383116, etc.), consisting of a complete open reading frame sequence with incomplete sequences of the 5′ and/or 3′ untranslated regions (UTRs). We therefore sought to determine whether our laboratory stocks of MR766 and H/PF/2013 matched the published sequences. MR766 is the prototype ZIKV strain, isolated in Uganda in 1947 (1), and has undergone extensive *in vivo* and *in vitro* passaging resulting in substrains. Conversely, H/PF/2013 is a human clinical isolate from French Polynesia, 2013, with limited passage in Vero cells (23). Our laboratory stocks of both of these viruses were grown in C6/36 cells, and cDNA was prepared from infected cell supernatant. Sanger sequencing was performed on PCR products to obtain a consensus sequence of the open reading frame, and these exactly matched deposited sequences for each virus (MR766, accession no. KU955594, and H/PF/2013, accession no. KJ776791). Of interest, we identified our MR766 strain as being among those substrains that carry a four-codon deletion that ablates a canonical N-linked glycosylation site in the envelope (E) glycoprotein (Fig. 1B), rather than an alternative MR766 substrain (and other ZIKV strains) that encodes a glycosylation site at this position (N154).

We next obtained complete sequences of the 5′ and 3′ genomic termini for both strains. We used a modified 5′-3′ rapid amplification of cDNA ends (RACE) method because conventional RACE protocols are dependent upon a 3′ poly(A) tail which is not present on flavivirus genomes (see Fig. S1A in the supplemental material). The deposited genomic sequence of MR766 included complete 5′ and 3′ termini, and our sequences matched these exactly (Fig. S1B and C). As the deposited sequence of H/PF/2013 consisted of a complete open reading frame with only partial UTR sequences, our analysis added 60 nucleotides (nt) to the 5′ end and 130 nucleotides to the 3′ end to generate a new complete genomic sequence for this virus (Fig. S1B and C). An additional contemporary outbreak strain, PRVABC59, a human clinical isolate from Puerto Rico, 2015, had the same 5′ and 3′ UTR sequences as H/PF/2013 except for a single difference in the 3′ UTR at nucleotide 10391 (Fig. S1B and C). Interestingly, the nucleotide change at position 10391 also occurs in both of the early Brazilian ZIKV isolates (SPH2015 and BeH819015) and matches the PRVABC59 sequence as well. This represents a diversity of only 0.23% between the 3′ UTRs of H/PF/2013 and PRVABC59, whereas the H/PF/2013 and MR766 3′ UTRs differ by 17 nucleotides (3.90%). These results indicate a high degree of conservation of the 5′ and 3′ UTR genomic regions, especially in natural clinical isolates that have not undergone extensive *in vitro* and *in vivo* passages.

10.1128/mBio.02014-16.1 FIG S1  Modified RACE protocol for determining flavivirus terminal genome sequences. Schematic representation of 5′-3′ RACE to sequence flavivirus genomic termini. Briefly, viral RNA (linear, single stranded) is treated with calf intestinal phosphatase (CIP) to remove terminal phosphates on uncapped RNAs that reduce downstream yields, particularly at the ligation step (A). The RNA is then decapped with tobacco acid pyrophosphatase (TAP), and the now-exposed genomic termini are ligated together with RNA ligase I. PCR primers flanking the ligated termini are used to generate a PCR amplicon for Sanger sequencing. The amplicon provides the sequence of both termini in a single reaction. (B) RACE sequencing of the 5′ termini of MR766 was performed as described above. (C) RACE sequencing of the 3′ termini of MR766 agreed with previously published results; however, 131 previously unpublished nucleotides were identified for PRVABC59, and 130 were identified in the 3′ UTR of H/PF/2013. Download FIG S1, TIF file, 0.7 MB. Copyright © 2017 Widman et al. 2017 Widman et al. This content is distributed under the terms of the Creative Commons Attribution 4.0 International license.

### Development of a ZIKV reverse genetics system using unidirectional assembly of a quadripartite genome.

We previously developed reverse genetics platforms that are comprised of multipartite genomes with unidirectional assembly to generate stable infectious clones for coronaviruses (24, 25) and DENV (20–22). Given the high genetic relatedness between DENV and ZIKV, we adapted this strategy to design a clone of our MR766 ZIKV substrain, which lacks E glycosylation due to a 4-amino-acid (aa) deletion (−Gly MR766), using a quadripartite subgenomic system to disrupt toxic genomic regions by partitioning them onto four high-copy-number plasmids for bacterial amplification (Fig. 1A and B). The partition sites were chosen to mimic those used to construct DENV infectious clones by this method (20) and by the location of naturally occurring class IIG restriction sites which cleave outside their recognition site, generating nonpalindromic ends that allow for unidirectional ligation of the purified four genomic fragments into full-length genomes. A T7 RNA polymerase promoter site was oriented directly upstream of the first ZIKV nucleotide and used for *in vitro* transcription of full-length ligated genomes, while a hepatitis delta virus ribozyme immediately following the last ZIKV nucleotide generates an authentic 3′ terminus on the full-length infectious genomic RNA (Fig. 1A). These transcripts were electroporated into C6/36 *Aedes albopictus* cells, which released high-titer infectious virus (approximately 10^6^ to 10^7^ focus-forming units [FFU]/ml) in the culture supernatants (Fig. 1D). These were collected and passaged once or twice on C6/36 cells to generate virus stocks used for experiments. To verify the presence of molecularly cloned viruses, we sequenced the full viral genome, confirming the genetic origins of each recombinant virus. Since the MR766 infectious clone included engineered ablation of duplicate restriction sites, we also digested cDNA from the clone and isolate viruses, confirming the expected restriction fragment sizes from two of the clones (Fig. 1E).

Although our substrain of MR766 exactly matched deposited sequences of this virus (accession number KU955594), other deposited MR766 substrain sequences differ and encode an intact N-linked glycosylation site at N154 of E. Since this N154 glycosylation site is a known determinant of virulence, neuroinvasion, and vector competence for other flaviviruses (26, 27), we constructed an additional MR766 variant with an intact glycosylation site based on this published sequence (accession number KU720415). A third clone restoring the 4-aa deletions but not encoding the N-linked glycosylation site was also generated (accession number LC002520). An amino acid alignment of the region surrounding the N154 glycosylation site highlights the differences in the clone viruses (Fig. 1B). Restoration of the 4-aa deletion and functional glycosylation was confirmed by Western blot analysis of E protein. As expected, glycosylated E migrates at the highest molecular weight followed by the +Del and −Gly clones and natural isolate (Fig. 1F). While peak infectious titers of all three clone viruses reached similar levels as our isolate virus with the 4-aa deletion (Fig. 1D), the viral clone with restored glycosylation appears to have an early growth advantage and to make larger infectious foci than the other allelic mutants (Fig. 1C; Fig. S2).

10.1128/mBio.02014-16.2 FIG S2  Infectious focus size of viruses. Individual foci on Vero cells 44 to 46 h postinfection were measured using a CTL Immunospot analyzer. At least 65 foci were measured per virus. (A) MR766 +Gly formed significantly larger foci than MR766 isolate, MR766 +Del, or MR766 −Gly by one-way ANOVA followed by Tukey’s multiple-comparison test. (B) H/PF/2013 isolate formed significantly larger foci than the infectious clone by unpaired *t* test. (C) ZIKV isolates formed significantly larger foci than BeH819015 or SPH2015 infectious clones by one-way ANOVA followed by Tukey’s multiple-comparison test. Download FIG S2, EPS file, 0.9 MB. Copyright © 2017 Widman et al. 2017 Widman et al. This content is distributed under the terms of the Creative Commons Attribution 4.0 International license.

**FIG 2:**
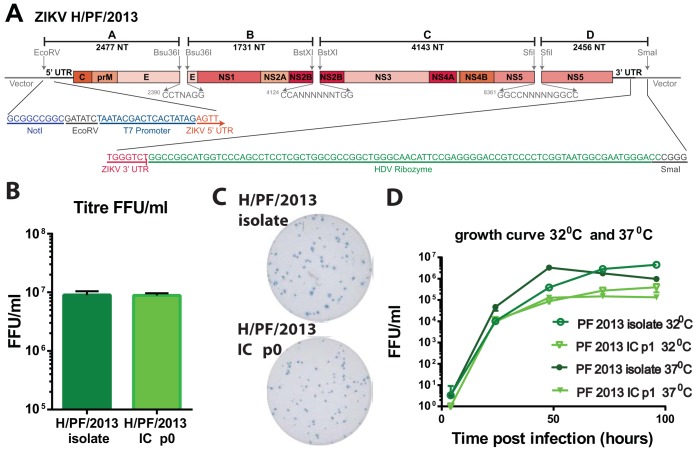
(A) Schematic diagram of ZIKV H/PF/2013 infectious clone. The genome of the virus is divided into 4 fragments using diagrammed restriction endonucleases and cloned into high-copy-number vectors. A T7 promoter and a hepatitis delta virus ribozyme flank the genome. Sizes of each fragment are shown. (B) H/PF/2013 virus isolate was harvested 4 days after infection of C6/36 cells at an MOI of 0.1. Recombinant virus was harvested 4 to 7 days after electroporation of RNA into C6/36 cells. Titrations were performed in triplicate. (C) Virus focus images on Vero-81 cells. (D) Growth curves of H/PF/2013 natural isolate and infectious clone at 32°C and 37°C. At both temperatures, the isolate grew significantly better than the infectious clone by 2-way analysis of variance.

Utilizing similar strategies, we constructed an infectious clone system for ZIKV strain H/PF/2013, considered to be a near-predecessor of the current outbreak in the Americas. Because of the genetic heterogeneity between MR766 and H/PF/2013, different restriction endonuclease sites were used in clone design (Fig. 2A). After electroporation of C6/36 cells, titers reached 10^6^ to 10^7^ FFU/ml, equivalent to that of the natural isolate (Fig. 2B), but the cells demonstrated slightly smaller infectious focus size and morphology (Fig. 2C; Fig. S2). We performed multistep growth curves on Vero-81 cells at two different temperatures to assess not only stability but also growth kinetics. The natural isolate and clone virus have similar growth kinetics at 32°C and 37°C (Fig. 2D), indicating that temperature had minimal impacts on the total amount of progeny virus that was produced during infection. As seen with other ZIKV recombinant viruses (16), peak infectious titers for the P1 stock of H/PF/2013 infectious clone (IC) were lower than those for the natural isolate at both temperatures. The likely differences in isolate and recombinant virus yields may reflect a loss in cooperativity due to the reduced quasispecies populations present in the molecularly cloned stocks (28, 29).

### Brazilian ZIKV strains.

**FIG 3:**
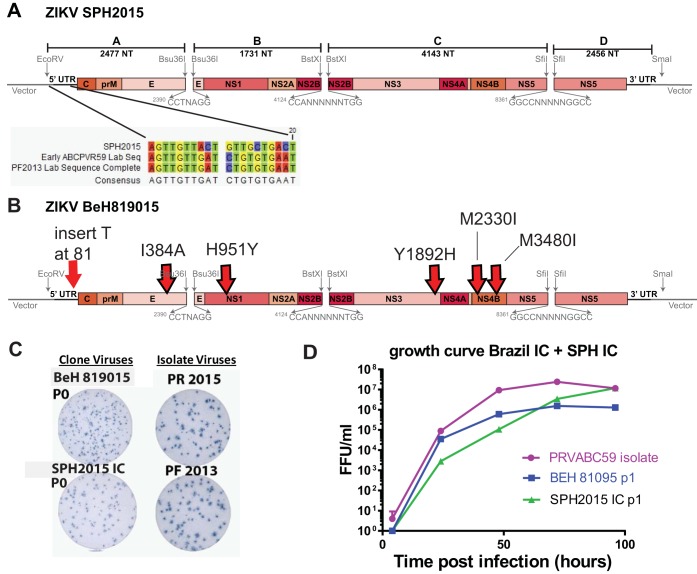
(A and B)** **Diagrams of ZIKV SPH2015 and BeH819015 infectious clones. (A) Diagram shows changes introduced into the first 20 nt to recover SPH2015 infectious clone virus. (B) Diagram of infectious clone BeH819015. Six nucleotide changes made to convert SPH2015 clone into BeH819015 are shown (red arrows). (C) Virus focus images on Vero-81 cells. (D) Growth curves of PRVABC59 natural isolate and infectious clones of SPH2015 and BeH819015 at 37°C. Although they differ by only 6 nucleotides, growth of SPH2015 was significantly different from that of BeH819015 by 2-way analysis of variance with Tukey’s test.

New ZIKV disease presentations, particularly microcephaly and other birth defects, were first observed during the 2015-2016 ZIKV outbreak in Brazil. In order to better assess the viral genetic determinants that may contribute to these emerging phenotypes, we sought to develop an infectious clone of a contemporary outbreak strain from Brazil. However, as we were unable to access any virus isolates from Brazil, we relied upon deposited sequences for clone construction. The first Brazilian ZIKV sequence published online was strain SPH2015 (accession number KU321639), which was isolated in March 2015 from a patient in São Paulo state (30) and predates more commonly used Brazilian ZIKV strains such as the December 2015 PRVABC59 isolate. While the 5′ UTR sequence of the SPH2015 strain was complete, the final 129 nucleotides of the 3′ UTR sequence were missing. Since we had no SPH2015 virus available for independent sequencing, we grafted the final 129 nucleotides from H/PF/2013 (which is identical to PRVABC59) onto the SPH2015 sequence to produce a complete ZIKV genome (Fig. 3A). The high nucleotide identity between H/PF/2013 and these early Brazilian strains enabled us to use identical junctions to construct the SPH2015 clone (and the BeH819015 clone [see below]), allowing easy production of chimeric viruses by assembling different permutations of subgenomic fragments.

After repeated failed attempts at recovery, we compared the deposited SPH2015 sequence to more recently published sequences and identified a series of seven nucleotides within the first 20 nt of the SPH2015 genome that did not match other ZIKV strains, two of which (nt 10 and 15) were different from all other flaviviruses (Fig. 3A). Using site-directed mutagenesis, we changed these seven nucleotides to match the sequences of H/PF/2013 and PRVABC59 (which are identical in this region). This modification allowed us to recover viable SPH2015 clone virus (Fig. 3C), suggesting that the published sequence contains lethal errors and highlighting the usefulness of our system in identifying and repairing such errors in the phylogenetic record.

### Identification of two genetically distinct clades of ZIKV circulating in Brazil.

**FIG 4:**
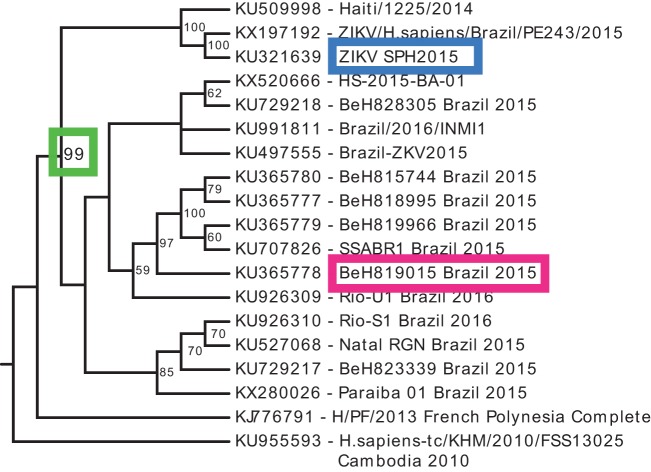
Phylogenetic tree of Brazilian ZIKV full-length sequences. ZIKV sequences were acquired from GenBank and this study (see Materials and Methods), and the 5′ UTR was removed from each lineage. Multiple sequence alignment was performed using MAFFT, and the phylogenetic tree was generated using maximum likelihood (RAxML software) with 100 bootstrap replicates. Only bootstrap support values of >50 are displayed at each node. GenBank accession number and strain name are indicated on each branch. Two distinct clades of Brazilian ZIKV sequences are highly supported (green box). SPH2015 and BeH819015 are boxed in blue and red, respectively.

Phylogenetic analysis of available ZIKV sequences from the Brazilian outbreak revealed the presence of two distinct clades of contemporary ZIKV strains (5); however, the specific structure of the phylogenetic tree is sensitive to sequence variations. Because the previously identified published 5′ UTR sequence of SPH2015 resulted in lethal variants, we excluded the 5′ UTR from our phylogenetic analysis, providing additional support for the existence of two independent clades of Brazilian strains (Fig. 4). The smaller clade is composed of two Brazilian sequences clustering with the Haiti/1225/2014 strain (Fig. 4), which preceded the first reports of ZIKV in Brazil. When the 5′ UTR sequences are included, the Haiti/1225/2014 clade is still a distinct monophyletic group; however, the specific topology and node confidence within each clade are slightly altered (Fig. S3). Irrespective of the phylogenetic approach (Fig. 4; Fig. S3), our infectious clones represent one isolate from each clade. The French Polynesian strain (H/PF/2013) acts as the closest relative to the Brazilian clades, consistent with previous phylogenetic analyses published using a wider geographic range of isolates (31–34).

**FIG 5:**
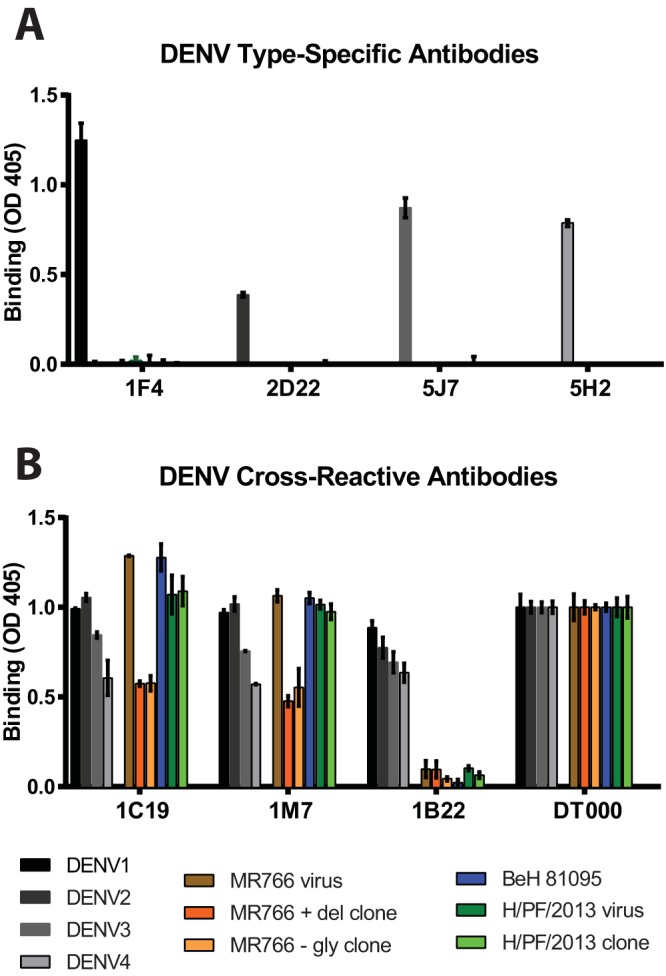
Antibody binding to DENV and ZIKV clone viruses. (A) The highly DENV serotype-specific MAbs 1F4, 2D22, 5J7, and 5H2 clearly bind only the appropriate DENV serotype but not ZIKV. (B) The DENV serotype-cross-reactive MAbs 1C19 and 1M7 bound all DENV and ZIKV strains tested, while 1B22, a prM-specific MAb, bound only the 4 DENV serotypes. DT000 is serum isolated from a traveler with repeat flavivirus vaccinations and infections and is used to control protein loading.

10.1128/mBio.02014-16.3 FIG S3  Phylogenetic trees of Brazilian ZIKV full-length sequences. ZIKV sequences were acquired from GenBank and this study (see Materials and Methods). (A) Sequences were modified to remove the 5′ UTR from each lineage. This was due to the inviability of Haiti/1225/2014 and ZIKV SPH2015 strains when the published 5′ UTR sequence was used. (B) Sequences including all published 5′ UTR sequences. (C) Sequences including the modified 5′ UTR sequences for Haiti/1225/2014 and ZIKV SPH2015 (Fig. 5A). Multiple sequence alignment was performed using MAFFT, and the phylogenetic tree was generated using maximum likelihood (RAxML software) with 100 bootstrap replicates. Only bootstrap support values of >50 are displayed at each node. GenBank accession number and strain name are indicated on each branch. Two distinct clades of Brazilian ZIKV sequences are highly supported in all three versions; however, the topology of each clade and support values are sensitive to the specific sequences used. Download FIG S3, TIF file, 1.8 MB. Copyright © 2017 Widman et al. 2017 Widman et al. This content is distributed under the terms of the Creative Commons Attribution 4.0 International license.

10.1128/mBio.02014-16.4 FIG S4  prM immunostaining. To evaluate whether DENV and ZIKV prMs are antigenically diverse, we immunostained infected monolayers with the well-characterized DENV-specific prM MAb 2H2, along with 4G2 as a control. In every case, the 4G2 was able to detect E in the infected monolayers, but 2H2 was able to detect prM only in the DENV-infected cells, suggesting antigenic variation. Download FIG S4, EPS file, 2.7 MB. Copyright © 2017 Widman et al. 2017 Widman et al. This content is distributed under the terms of the Creative Commons Attribution 4.0 International license.

Our modified SPH2015 sequence falls into the smaller clade of Brazilian ZIKV. One of the earliest and most complete sequences that we identified from the larger clade was strain BeH819015 (accession number KU365778), which was isolated in Pará state in July 2015 (5). The nucleotide sequence of BeH819015 is closest to but differs from the ZIKV strains clustering with Haiti/1225/2014, from isolates from Oceania (such as H/PF/2013), and from other isolates from the Caribbean (such as PRVABC59). To demonstrate the tractability of our reverse genetics platform, we generated an infectious clone of ZIKV BeH819015 by using site-directed mutagenesis to change the six nucleotide differences (five nonsynonymous changes and one nucleotide insertion in the 5′ UTR) in SPH2015 to those of BeH819015 (Fig. 3B). The BeH819015 recombinant was viable and made infectious foci of similar size as the SPH2015 IC, but both are smaller than H/PF/2013 and PR2015 (Fig. 3C; Fig. S2). Because we had no actual Brazilian virus in the lab with which to compare our clones, we used the abovementioned PVRABC59 as a control for comparing the growth kinetics of our Brazilian recombinant viruses *in vitro*. As shown in Fig. 3D, BeH819015 has a lower peak titer and SPH2015 has slightly slower early growth; however, our synthesized Brazilian clones do not have growth kinetics vastly altered from those of a commonly used natural isolate strain. Taken together, these results demonstrate that we have reproduced a time-ordered family of infectious ZIKV molecular clones and recombinant viruses that precede and encompass the pandemic in the Western Hemisphere (Fig. 4, blue and red boxes).

### Antigenicity of ZIKV clones and cross-reactivity with DENV.

We used a virus capture enzyme-linked immunosorbent assay (ELISA) to measure binding of a panel of DENV monoclonal antibodies (MAbs) to the panel of isolate and recombinant ZIKVs, compared to the four DENV serotypes. The MAb panel included four DENV serotype-specific neutralizing MAbs (1F4, 2D22, 5J7, and 5H2) (Table S1). As expected, none of these MAbs bound to any of the ZIKV strains tested (Fig. 5A). In contrast, MAbs 1C19 and 1M7, which bind the highly conserved fusion-loop region of all DENV serotypes (Table S1), bound to all tested ZIKV strains (Fig. 5B). Dissimilarly, MAb 1B22, which binds to prM on immature DENV virions, bound to all four DENV serotypes but none of the ZIKV strains tested (Fig. 5B). To determine whether the lack of 1B22 binding to ZIKV was due to the absence of prM present on ZIKV virions (35), or antigenic divergence between DENV and ZIKV prM, we infected Vero-81 cells with DENV4 or ZIKV and stained permeabilized cells. While anti-fusion loop MAb 4G2 stained all infected cells, anti-prM DENV MAb 2H2 stained only DENV4-infected cells (Fig. S3), likely reflecting antigenic divergence in prM between DENV and ZIKV. As there is extensive cross-reactivity between antiflavivirus immune sera and ZIKV (36, 37), defining cross-reactive and virus-specific epitopes between ZIKV and DENV is a critical component of developing ZIKV diagnostics and understanding the mechanisms of ZIKV pathogenesis.

10.1128/mBio.02014-16.5 TABLE S1  MAbs used in binding ELISA. Download TABLE S1, DOCX file, 0.01 MB. Copyright © 2017 Widman et al. 2017 Widman et al. This content is distributed under the terms of the Creative Commons Attribution 4.0 International license.

### ZIKV infectious clones are virulent in a mouse model of lethal ZIKV disease.

**FIG 6:**
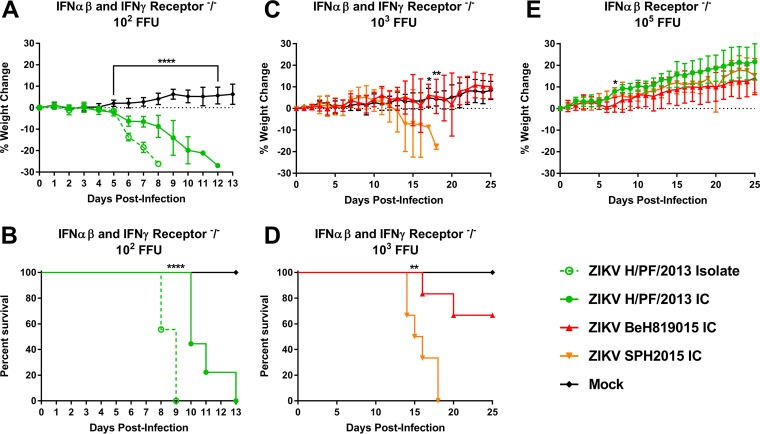
*In vivo* pathogenesis studies. ZIKV H/PF/2013 isolate (*n* = 9), infectious clone virus (*n =* 9), or PBS (*n =* 7) was inoculated into IFNGR-knockout mice by footpad inoculation with 10^2^ FFU (A and B). In parallel, *Ifnar1*^−/−^ and *Ifngr1*^−/−^ mice were inoculated by footpad injection with 10^3^ FFU of BeH819015 (*n =* 6) or SPH2015 (*n =* 6) infectious clone or PBS (*n =* 3) (C and D) or 10^5^ FFU of recombinant SPH2015 (*n =* 5), BeH819015 (*n =* 5), or H/PF/2013 (*n =* 5) infectious clone (E), respectively. Weight loss and mortality were recorded through 25 days postinfection or until all animals had succumbed to infection.

Previous work has demonstrated that wild-type immunocompetent mice are resistant to ZIKV pathogenesis but that mice lacking alpha/beta interferon (IFN-α/β) signaling (e.g., *Ifnar1*^−/−^, A129, AG129, *Irf3*^−/−^ × *Irf5*^−/−^ × *Irf7*^−/−^) succumb to ZIKV infection (38, 39). We infected 6- to 8-week-old *Ifnar1*^−/−^ × *Ifngr1*^−/−^ (C57BL/6) mice with 10^2^ to 10^3^ FFU of ZIKV by a subcutaneous route and monitored weight loss and lethality daily for 14 days (Fig. 6A to D). Mice were infected with the ZIKV H/PF/2013 isolate (*n* = 9) or the H/PF/2013 infectious clone (*n* = 9) or were mock infected with phosphate-buffered saline (PBS) (*n =* 7). Mice that received H/PF/2013 virus began to lose weight at 5 days postinfection (Fig. 6A), while H/PF/2013 clone-infected mice exhibited more gradual weight loss than those infected with the isolate virus (*P* < 0.0001). All ZIKV-infected mice succumbed to infection (Fig. 6B), but there was a statistical difference by log rank for recombinant viruses. Additional mice were infected with either of the Brazilian clones (SPH2015 IC or BeH819015 IC) with a higher dose of 10^3^ FFU due to unpublished data suggesting that these infectious clones are attenuated in comparison to the H/PF/2013 viruses in mice. SPH2015-infected mice started losing weight by day 10 (Fig. 6C), signs of illness appeared by day 12, and all mice succumbed to illness by day 18 (Fig. 6D). A statistical difference between the infected groups was seen at days 17 and 18 by Bonferroni test (*P* = 0.0184 and 0.009, respectively). Sixty-six percent of BeH819015-infected mice showed no overt disease signs, leading to a statistical difference in lethality when measured by a log-ranked Mantel test between strains (Fig. 6D). In contrast, when we infected 6- to 8-week-old *Ifnar1*^−/−^ mice with 10^5^ FFU of ZIKV (clones SPH2015, H/PF/2013, and BeH819015), we observed no signs of illness or weight loss over the course of 25 days (Fig. 6E). The increased morbidity and mortality in *Ifnar1*^−/−^ × *Ifngr1*^−/−^ mice compared to *Ifnar1*^−/−^ mice for all viruses tested reveal a specific role for gamma interferon (IFN-γ) in controlling ZIKV pathogenesis.

## DISCUSSION

The emergence of ZIKV is the latest example of an Old World arbovirus spreading rapidly upon arrival in the Americas, reminiscent of Chikungunya virus in 2013, West Nile virus in 1999, multiple DENV serotypes in the 1980s, and, originally, the introduction of yellow fever virus in the 17th century. The emergence of ZIKV in the Americas and the concomitant association with congenital infection highlight the importance of understanding the fundamental molecular mechanisms regulating flavivirus transmission, spread, and emergence. Here, we report the generation of a ZIKV reverse genetics system, representing strains from both the African and Asian ZIKV lineages. We used synthetic approaches to recover two recombinant viruses (SPH2015 and BeH819015) representing early epidemic strains from two contemporary clades of ZIKV that are circulating in Brazil. This panel of infectious clones also includes three allelic variants of the prototype ZIKV strain (MR766). Additionally, we generated a clone of an isolate from French Polynesia (H/PF/2013) that immediately preceded the current epidemic in the Americas and is likely representative of the ZIKV strain that was introduced to Brazil.

Several groups have reported ZIKV clones using low-copy-number plasmids, plasmids with introns, or multipiece systems (15–17, 19) to overcome the bacterial toxicity of the plasmids. Shan et al. derived a full-length infectious cDNA clone of a 2010 ZIKV strain from Cambodia (FSS13025) using a sequentially assembled multipiece system to generate a single-piece infectious clone (40). FSS13025 falls within the Asian lineage of ZIKV strains but differs by approximately 19 amino acids from Latin American strains such as SPH2015 and BeH819015. Other infectious clones of ZIKV strain Paraiba_01/2015 (16) and MR766 (15) were propagated in a bacterial artificial chromosome as complete genomes containing engineered introns and launched from DNA, not RNA. Using our system, we generated a clone of the BeH819015 virus by introducing six mutations to the SPH2015 background, which was straightforward because the changes were distributed across the subgenomic fragments on four plasmids. In contrast to other systems (16, 17), all of our subgenomic regions are maintained in high-copy-number plasmids. Our quadripartite assembly scheme allows for flexible construction of chimeric viruses, as contemporary strains share sufficient sequence homology to use identical restriction sites, and so subgenomic fragments from different strains can be interchanged. This allows rapid mapping of genetic determinants between two virus strains. An overlapping quadripartite system was recently used to construct a different MR766 strain incorporating green fluorescent protein (GFP) (19); however, the stability of these reporter viruses was unclear given that GFP flaviviruses are generally unstable (41).

Previous studies have analyzed ZIKV sequences from the current epidemic (5, 34, 42, 43), and two clades can be seen in some analyses; however, these reports have not discussed the significance of the two distinct clades of Brazilian ZIKV isolates, as was also found in our analysis (5, 34, 42, 43). The presence of two distinct clades could indicate independent introductions of ZIKV into Brazil, an earlier introduction than previously appreciated, novel evolutionary patterns, or errors in the published sequences. Additional factors to consider include geographic location, tissue source, and/or disease presentation of the isolate viruses, although no relationship between these factors and the clade separation was evident in the sequences considered in our analysis. Better resolution of the tree topology and potential characteristics defining each clade will be achieved as more isolates are sequenced and incorporated into phylogenetic analyses. We generated infectious clones of two Brazilian ZIKV strains representing each of the two identified clades, which will facilitate further analysis of the properties that distinguish these two groups of epidemic ZIKV strains.

The ZIKV virus clones from both Brazilian clades (SPH2015 and BeH819015) are antigenically similar to the Asian strain (H/PF/2013) and the African strain (MR766), consistent with the idea that ZIKV strains circulate as a single serotype. DENV serotype-specific antibodies do not bind any of the ZIKV isolates or clones, confirming the unique antigenic areas of E protein between DENV and ZIKV. Conversely, all ZIKV strains are recognized by cross-reactive DENV fusion-loop antibodies, affirming the homology of ZIKV and DENV in this region. In contrast to the substantial antigenic cross-reactivity between ZIKV and DENV E protein, a MAb that recognizes prM of all four DENV serotypes did not bind to cells infected with any ZIKV strains, suggesting that prM is not antigenically conserved. Currently, available ZIKV diagnostics are confounded by cross-reactive binding to DENV in standard ELISAs (36, 37), so identifying antigenically distinct epitopes is important for developing diagnostics that can identify ZIKV infection in DENV-seropositive individuals.

In *Ifnar1*^−/−^ × *Ifngr1*^−/−^ mice, the H/PF/2013 isolate was significantly more virulent than recombinant H/PF/2013, likely reflecting the reduced quasispecies complexity of P0 recombinant virus stocks (16), a known virulence determinant (28, 29). Although the BeH819015 and SPH2015 recombinant viruses differ by only six nucleotide changes throughout their genomes, SPH2015 infection resulted in 100% mortality and earlier morbidity than the less pathogenic BeH819015 strain. Importantly, all three recombinant viruses were attenuated in *Ifngr1*^−/−^ mice, demonstrating the critical importance of IFN-γ responses in controlling lethal ZIKV infections, as also reported by other groups (38). It is interesting that the northern Brazil BeH819015 isolate was more strongly associated with severe congenital disease than SPH2015 (5) but more attenuated in the mouse model. Additional studies will be needed to identify the key residues responsible for reduced BeH819015 pathogenesis *in vivo*.

After ZIKV infection, an unexpected development is the severe disease presentations, most significantly congenital infection and birth defects, associated with the current ZIKV outbreak in Latin America and the Caribbean (44) and replicated in mouse models of human disease (45, 46). An association between ZIKV infection and Guillain-Barré syndrome was first noted during the 2013-2014 outbreak in French Polynesia (47), and retrospective analyses of this outbreak identified previously unappreciated associations with fetal neurodevelopmental abnormalities (48). More recently, ZIKV-associated Guillain-Barré syndrome has been reported in the Americas as well (49, 50). It is unclear why severe manifestations of ZIKV infection have become evident only in the most recent outbreaks, and possible explanations include host genetic background, host immune status, and the large size and intense surveillance of the current epidemic. However, it is plausible that viral genetic changes could result in new disease phenotypes. Previous analyses have speculated that this may be the case, based on sequence comparisons of historic and contemporary ZIKV strains (42, 43). However, testing this hypothesis requires the ability to generate isogenic mutants to evaluate viral determinants of pathogenesis. Our panel of ZIKV infectious clones representing historical and contemporary virus strains, as well as the relative ease of generating allelic variants and chimeric viruses using the quadripartite system, provides an essential toolkit for determining the mechanisms of ZIKV pathogenesis and furthering the development of new vaccines and antivirals.

## MATERIALS AND METHODS

### Cells and viruses.

The MR766 strain of ZIKV was obtained from the World Reference Center for Emerging Viruses and Arboviruses (R. Tesh, University of Texas Medical Branch). ZIKV strains H/PF/2013 and PRVABC59 were provided by the U.S. Centers for Disease Control and Prevention. Virus stocks were prepared in C6/36 *Aedes albopictus* cells (ATCC catalog no. CRL-1660) or, where indicated, Vero-81 *Cercopithecus aethiops* cells (ATCC catalog no. CCL-81) and titrated in Vero-81 cells (51). C6/36 cells were grown at 32°C with 5% CO_2_ in Eagle’s minimum essential medium (MEM) supplemented with 5% heat-inactivated (HI) fetal bovine serum, nonessential amino acids, and antibiotics/antimycotics. Vero-81 cells were grown at 37°C with 5% CO_2_ in Dulbecco’s modified Eagle’s medium (DMEM)–F-12 50/50 medium (Gibco) supplemented with 5% HI fetal bovine serum and antibiotics/antimycotics (52). For growth curve analyses, cells were infected at a multiplicity of infection (MOI) of 0.1 in triplicate, and supernatants were collected at 4, 24, 48, 72, and 96 h postinfection and frozen at −80°C until titrated on Vero-81 cells. Viral foci were detected at 44 to 48 h after infection, following fixation/permeabilization with 4% paraformaldehyde-0.01% saponin using primary MAb E60 (38) or 4G2 (51) and secondary horseradish peroxidase (HRP)-conjugated goat anti-mouse IgG (Sigma), followed by TrueBlue substrate (KPL). Number and size of foci were analyzed with a CTL Immunospot instrument.

### Viral genome sequencing and modified 5′-3′ RACE.

Viral RNA was isolated using a QIAamp viral RNA minikit (Qiagen), and cDNA libraries were prepared using Superscript III (Invitrogen). Sanger sequencing was performed on PCR templates generated using Phusion High-Fidelity DNA polymerase (New England BioLabs) and analyzed using Sequencher (Gene Codes) and Lasergene (DNAStar) software.

The 5′ and 3′ UTR sequences were determined as previously described (53). Briefly, viral RNA was isolated and treated with calf intestinal phosphatase (Ambion) for 1 h at 37°C to remove terminal phosphates of uncapped RNAs. Following a phenol-chloroform extraction and isopropanol precipitation, the RNA was treated with tobacco acid pyrophosphatase (Ambion) for 1 h at 37°C. After another phenol-chloroform extraction and isopropanol precipitation, the RNA was incubated with T4 RNA ligase I (Ambion) for 1 h at 37°C and then overnight at 4°C, to ligate the 5′ and 3′ ends. cDNA (Superscript III; Invitrogen) was made and used to generate an amplicon containing both the 5′ and 3′ UTR sequences, which was Sanger sequenced.

### ZIKV infectious clone design.

We designed a quadripartite unidirectional molecular clone strategy similar to that previously described for DENV and emerging coronaviruses (20–22, 24, 25). First, we identified naturally occurring class IIG nonpalindromic restriction endonuclease sites within the six ZIKV full-length genomes. For the clone of strain MR766, synonymous nucleotide changes were introduced at positions 895, 901, 2983, 7834, 7837, 8467, and 8473 to eliminate four internal restriction enzyme sites (two PflMI sites, an SmaI site, and a BstAPI site, respectively), simultaneously leaving marker mutations for identifying recombinant viruses. A T7 promoter sequence was added to the immediate 5′ end of the genome, and a hepatitis delta virus ribozyme was added directly after the last nucleotide of each ZIKV genome to generate an authentic 3′ end. The four subgenomic fragments were synthesized into the pUC57 vector (BioBasic) and amplified in *Escherichia coli* strain MC1061. The resulting purified plasmids were digested, ligated, *in vitro* transcribed, and electroporated into C6/36 cells as previously described (20–22). Supernatants from electroporated C6/36 cells were harvested after 6 to 7 days and passaged once on C6/36 cells to generate virus stocks. Clone MR766 +Del and MR766 +Gly viruses were generated by site-directed mutagenesis of the MR766 −Gly clone. Except for the junction sites (Bsu36I, BstXI, and SfiI), we used an identical strategy as for the H/PF/2013 clone and for SPH2015. Strain BeH819015 was generated by site-directed mutagenesis of the SPH2015 clone in the six positions where the two strains differed (5′ UTR, T81ins; E, I384V; NS1, H951Y; NS3, Y1892H; NS4B, M2330I; and NS4B, M2480I). We confirmed the sequence of all recombinant viruses.

### Phylogenetic analysis.

Full-length sequences (>10,000 nt) were obtained from GenBank and from sequencing of laboratory clones/isolates. Sequences were either manually edited to remove the 5′ UTR, left as downloaded, or manually modified to provide the viable 5′ UTR sequence variants for Haiti/1225/2014 and SPH2015. Multiple sequence alignments were performed using MAFFT (54). The best substitution model for each alignment was evaluated using jModelTest (55) and identified to be a general time-reversible (GTR) model with an estimated proportion of invariable sites and estimated gamma shape parameter (GTR + I + G). Maximum likelihood phylogenetic trees were generated using RAxML (56) using 100 bootstrap replicates. Trees were visualized using EvolView (57).

### Virus capture ELISA.

Virus particles were captured using mouse anti-DENV MAbs 4G2 and 2H2 in carbonate buffer. MAbs (see Table S1 in the supplemental material) were diluted to a concentration of 20 ng/µl and added to captured virus for 1 h at 37°C. After incubation with alkaline phosphatase-conjugated secondary antibodies (Sigma), *p*-nitrophenylphosphate substrate (Sigma) was added and absorbance at 405 nm was measured (Bio-Rad). Background signal (optical density at 405 nm [OD_405_] with no primary antibody) was subtracted from each virus sample, and absorbance was normalized to binding of an antiflavivirus human polyclonal serum sample.

### Animal studies.

Animal husbandry and experiments were performed under the approval of the University of North Carolina at Chapel Hill Institutional Animal Care and Use Committee. Six- to 8-week-old male and female *Ifnar1*^−/−^ × *Ifngr1*^−/−^ mice on a C57BL/6 background were infected subcutaneously via a footpad injection with 10^2^ FFU of ZIKV H/PF/2013 isolate, infectious clone virus, or PBS. In a separate study, mice were infected with 10^3^ FFU of BeH819015 or SPH2015 infectious clones or PBS. Mice were monitored daily for signs of morbidity or mortality and twice daily after losing 20% of their starting weight. Animals that exhibited dual hind limb paralysis or loss of 30% of their starting weight or that became moribund were humanely euthanized. Experiments were terminated 5 days following the last signs of illness resolving. Six- to 8-week-old male and female *Ifnar1*^−/−^ mice (C57BL/6) were infected with 10^5^ FFU of ZIKV clones of SPH2015, BeH819015, or H/PF/2013. Mice were monitored for 25 days with no adverse events noted.
