# Supplementary figures and images for: A Reverse Genetics Platform That Spans the Zika Virus Family Tree
Source: mBio. 2017 Mar 7;8(2):e02014-16. doi: 10.1128/mBio.02014-16 (PMC5340872; doi:10.1128/mBio.02014-16)

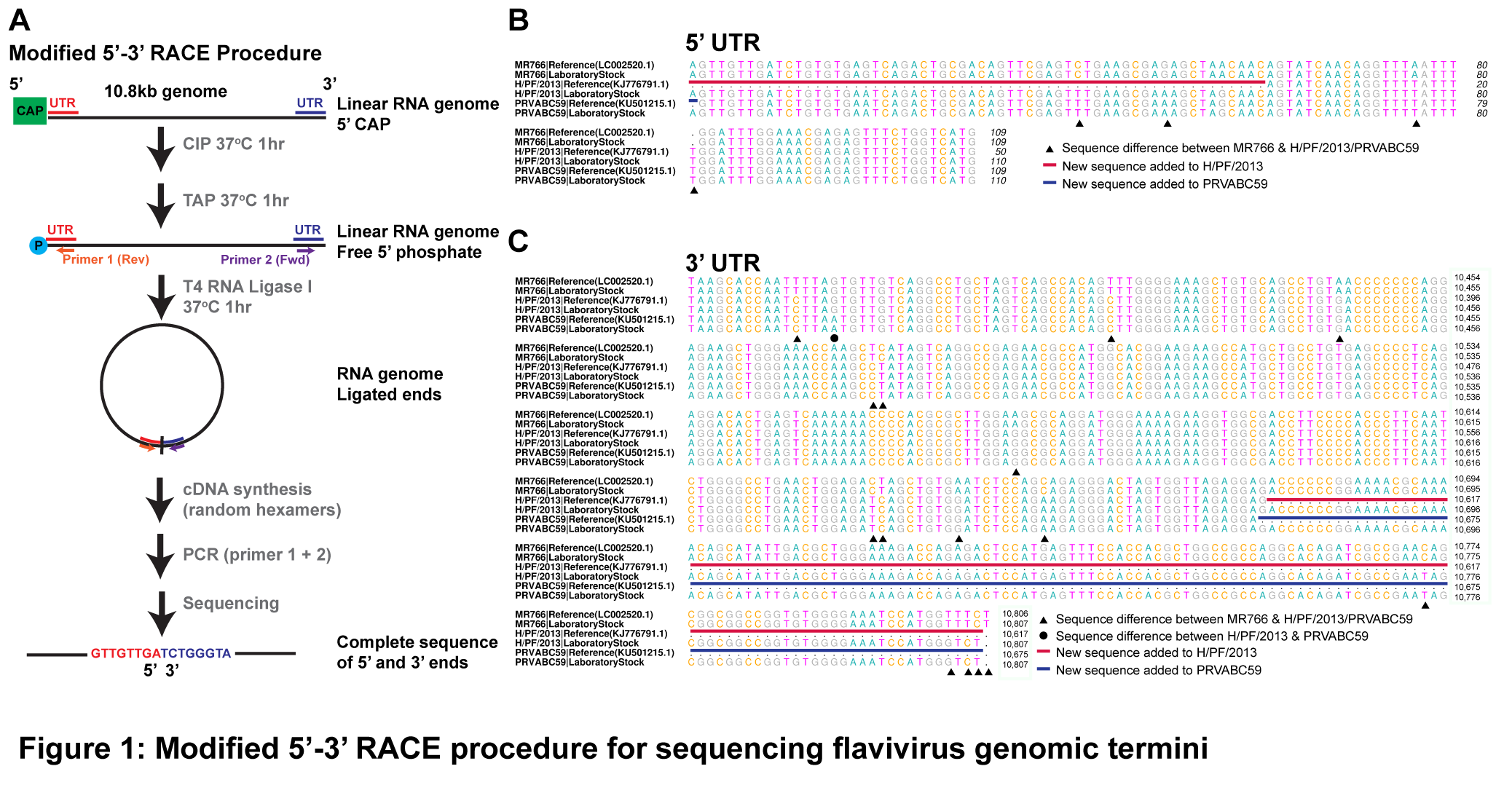

Supplement: FIG S1 [file mbo001173228sf1.tif]

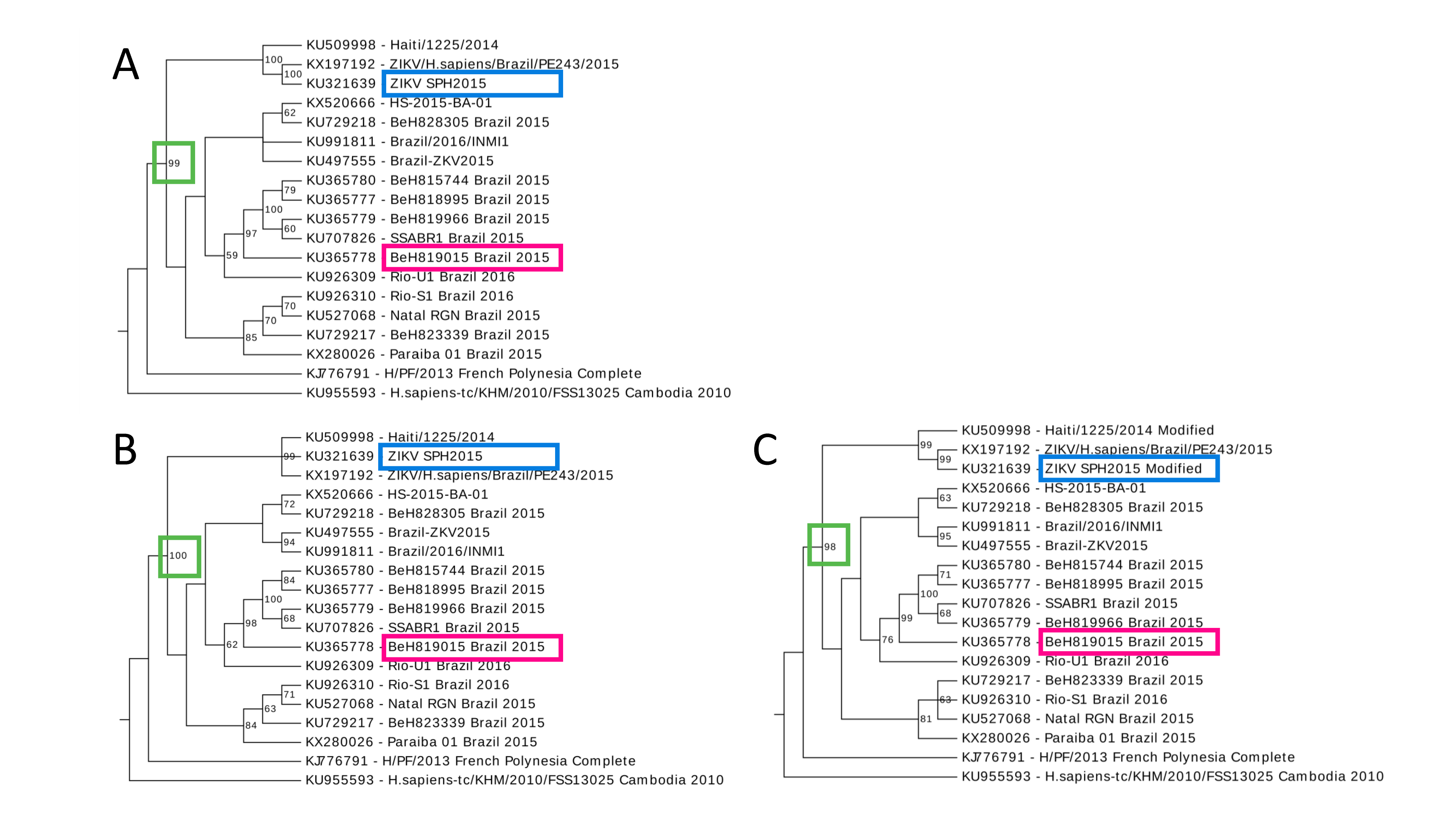

Supplement: FIG S3 [file mbo001173228sf3.tif]
