# Supplementary material for: A Reverse Genetics Platform That Spans the Zika Virus Family Tree
Source: mBio. 2017 Mar 7;8(2):e02014-16. doi: 10.1128/mBio.02014-16 (PMC5340872; doi:10.1128/mBio.02014-16)
Supplement: TABLE S1 [file mbo001173228st1.docx]

**Supplemental Table 1. MAbs used in binding ELISA**

| **Type** | **MAb** | **Epitope** | **Reference** |
| --- | --- | --- | --- |
| DENV1 type-specific | 1F4 | EDI | https://www.ncbi.nlm.nih.gov/pubmed/24421336 |
| DENV2 type-specific | 2D22 | EDII: fusion-loop | https://www.ncbi.nlm.nih.gov/pubmed/26138979 |
| DENV3 type-specific | 5J7 | EDI:EDII hinge | https://www.ncbi.nlm.nih.gov/pubmed/25698059 |
| DENV4 type-specific | 5H2 | EDI | https://www.ncbi.nlm.nih.gov/pubmed/22139356 |
| DENV cross-reactive | 1B22 | prM | https://www.ncbi.nlm.nih.gov/pubmed/25275316 |
|  | 1M7 | EDII fusion loop region | https://www.ncbi.nlm.nih.gov/pubmed/24255124 |
|  | 1C19 | EDII fusion loop region |  |
